# CAERUS: Predicting CAncER oUtcomeS Using Relationship between Protein Structural Information, Protein Networks, Gene Expression Data, and Mutation Data

**DOI:** 10.1371/journal.pcbi.1001114

**Published:** 2011-03-31

**Authors:** Kelvin Xi Zhang, B. F. Francis Ouellette

**Affiliations:** 1 Graduate Program in Bioinformatics, University of British Columbia, Vancouver, British Columbia, Canada; 2 Ontario Institute for Cancer Research, Toronto, Ontario, Canada; 3 Department of Biological Chemistry, David Geffen School of Medicine, University of California Los Angeles, Los Angeles, California, United States of America; 4 Howard Hughes Medical Institute, David Geffen School of Medicine, University of California Los Angeles, Los Angeles, California, United States of America; Dana-Farber Cancer Institute, United States of America

## Abstract

Carcinogenesis is a complex process with multiple genetic and environmental factors contributing to the development of one or more tumors. Understanding the underlying mechanism of this process and identifying related markers to assess the outcome of this process would lead to more directed treatment and thus significantly reduce the mortality rate of cancers. Recently, molecular diagnostics and prognostics based on the identification of patterns within gene expression profiles in the context of protein interaction networks were reported. However, the predictive performances of these approaches were limited. In this study we propose a novel integrated approach, named CAERUS, for the identification of gene signatures to predict cancer outcomes based on the domain interaction network in human proteome. We first developed a model to score each protein by quantifying the domain connections to its interacting partners and the somatic mutations present in the domain. We then defined proteins as gene signatures if their scores were above a preset threshold. Next, for each gene signature, we quantified the correlation of the expression levels between this gene signature and its neighboring proteins. The results of the quantification in each patient were then used to predict cancer outcome by a modified naïve Bayes classifier. In this study we achieved a favorable accuracy of 88.3%, sensitivity of 87.2%, and specificity of 88.9% on a set of well-documented gene expression profiles of 253 consecutive breast cancer patients with different outcomes. We also compiled a list of cancer-associated gene signatures and domains, which provided testable hypotheses for further experimental investigation. Our approach proved successful on different independent breast cancer data sets as well as an ovarian cancer data set. This study constitutes the first predictive method to classify cancer outcomes based on the relationship between the domain organization and protein network.

## Author Summary

It is widely known that cancer is a complex process in which a large number of genes appear to be involved. Through experimental approaches, some oncogenes and tumor suppressors have been identified as playing important roles in the signaling and the regulatory pathways. However, we have not fully understood the complete mechanism of how cancer develops and how it leads to different disease outcomes (aggressive/dangerous or non-aggressive/less-dangerous). In order to identify a list of gene signatures and better predict cancer outcome, we developed an integrated and systematical approach by investigating gene expression profiling alternation caused by disruptions between protein-protein interactions and domain-domain interactions in the human interactome. Our approach achieves the favorable predictive performance if tested on a set of well-documented breast cancer patients, which suggests that the disrupted interactome is important to determine patient prognosis. Our approach is robust if tested on other independent data sets. This work provides a promising prognostic tool to classify different cancer outcomes.

## Introduction

Cancer development is a complex process driven by multiple genetic and environmental factors [1], [2], [3]. Understanding the underlying mechanism of this process and identifying related markers to assess the outcome of this process could lead to better management and treatment of this complex disease. For example, the majority of breast cancer patients are currently over-treated [4] due to the lack of accurate assessment of the risk of metastasis. As a result, a substantial proportion of patients are receiving the otherwise avoidable aggressive adjuvant therapy in accordance to the current guidelines [5]. Although the importance of identifying prognostic signatures that could accurately predict cancer outcomes is widely appreciated, it has remained a challenging task. With the emergence of large amounts of DNA microarray-based tumor gene expression profiles, molecular diagnostics and prognostics have begun to provide solutions to this challenge [6]. Several predictive tools [7], [8], [9], [10] were reported to classify different cancer outcomes primarily based on the identification of gene expression signatures observed in these outcomes. However, the predictive performance of these approaches was limited. For instance, in two large-scale expression studies [9], [10], approximately 70 gene markers were identified that could be used in the prediction of the metastasis in breast cancer, but only with an accuracy of 60–70%. This relatively low accuracy could be explained by some intrinsic shortcomings of the microarray data, as different experiment and analysis designs could yield inconsistent results due to systematic errors [11] and by the heterogeneity of carcinogenesis resulting from multiple factors such as specific samples and cancer types [6]. Recently, the prognostic predictive performance has been improved by integrating the gene expression profiles and the human interactome data, based on the notion that disruption of protein interaction network might affect disease outcomes [12]. Protein-protein interactions (PPIs) play an important role in the process of carcinogenesis. At the molecular level, any genetic alternation such as somatic mutations, translocations, deletions and insertions that modify expressed protein-coding genes could cause changes in a PPI-based regulatory mechanism that governs normal cell function. This could lead to aberrant or uncontrolled cell growth and eventually to cancer [1]. For example, mutations in the zinc finger domain presented in the oncoprotein MDM2 can disrupt the interaction of MDM2 with ribosomal proteins L5 and L11 and mediate p53 degradation [13]. The recent availability of large-scale PPI networks has made it possible to identify better gene signatures by combining the gene expression measurements with the perturbed protein interaction networks in the cell. Chuang and colleagues [14] developed a method to find subnetwork-based signatures by incorporating PPI networks and gene expression profiles. The resultant subnetworks with their gene expression profiles were used as markers to predict the prognosis of breast cancer patients. This study yielded an accuracy of 70–72% in determining a breast cancer as metastatic versus non-metastatic. Their study revealed the usefulness of the PPI network in conjunction with the gene expression profiles and provided a starting point to future studies. More recently, Taylor and colleagues [12] proposed a new methodology to predict breast cancer outcome based on the correlation of gene expression profiles between hub proteins and their interacting partners in the PPI network. This approach showed improved predictive performance at an accuracy of 76% when tested on a different set of gene expression profiles from breast cancer patients. These studies demonstrated that the topology of a PPI network could be a helpful line of biological evidence in differentiating cancer outcomes. In the meantime, however, there are other important biological elements that might be involved in the development of cancer genome and phenotype. To further strengthen the power of novel predictive tools, these lines of biological evidence need to be investigated and incorporated if proven useful.

**Figure 1:**
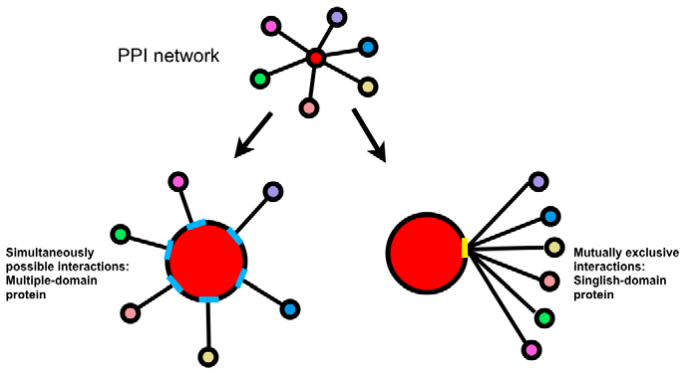
A schematic view of a ‘singlish-interface’ protein and a ‘multiple-interface’ protein. Given a protein (red node) and its neighboring proteins in the protein interaction network, we can define it as a ‘singlish-interface’ protein or a ‘multiple-interface’ protein. The ‘singlish-interface’ protein interacts with its neighboring proteins through the same domain (the yellow line); therefore, those domain-domain interactions are mutually exclusive. Conversely, the ‘multiple-interface’ protein interacts with its neighboring proteins through different domains (blue lines), as those interactions are simultaneously possible.

In an alternative approach, we focused on the prediction of cancer outcomes within the context of domain interaction network. Domains are defined as independent structure and/or functional blocks of proteins. It is clear that protein-protein interactions are mediated by the interactions between protein domains [15]. For example, SH2 domains mediate many critical protein interactions in signal transduction [16], [17]. Disrupted domain-domain interactions (DDIs) have been shown to stop the chain reaction of biological pathways at any point [18], [19], thus lead to various diseases [20], [21], [22]. This fact has motivated us to investigate the disruptions in a PPI network that are caused by DDIs, which might be a defining feature of tumor phenotype and thus could be used to determine patient prognosis. In the context of DDIs, we can categorize a given interacting protein into one of the two types based on the relationship of this protein and its neighboring proteins in the protein interaction network (Figure 1). We call a protein a ‘singlish-interface’ protein if it interacts with its neighboring proteins through the same domain-domain interaction; therefore, those domain-domain interactions are mutually exclusive (Figure 1A). Conversely, we call this protein a ‘multiple-interface’ protein if it interacts with its neighboring proteins through different domain-domain interactions, as those interactions are simultaneously possible (Figure 1B). It has been demonstrated that singlish-interface proteins evolve faster than multiple-interface proteins and are more likely to interrupt protein interactions and disturb the protein interaction network [23]. Therefore, we hypothesize that singlish-interface proteins are also more likely to be involved in the process of tumor progression than multiple-interface proteins. Meanwhile, DDIs could be interrupted by genomic variations located within interacting domains. One type of these genomic variations is somatic mutation. Somatic mutations are genetic alternations in DNA that are neither inherited nor passed to offspring. Some of these are thought to be driving the cancer process and have been refereed to as “driver mutations”, which can contribute to the development of the cancers or other diseases [24]. Therefore, we sought to investigate the perturbation of the protein interaction network in cancerous cells caused by the presence of somatic mutations, and to examine whether somatic mutation data could provide help in the prediction of cancer outcome. In summary, in addition to PPI data and gene expression data, we looked into incorporating two other types of data that might be functionally associated to the disturbance of the PPI networks: domain-domain interactions (DDIs) and somatic mutations.

In this study, we propose an integrated approach, named CAERUS, to predict the likelihood of cancer outcomes in unknown cancer patients provided the gene expression profiles of these patients are available. To implement CAERUS, we first developed a model to score each protein present in the expression profiles based on the domain connections to their interacting partners and the somatic mutations located in the domains. Next, gene signatures defined as proteins whose scores are above a preset threshold were identified. Then we computed the correlation of gene expression profiles of the gene signatures and their neighboring proteins. A modified naïve Bayes classifier was used to predict cancer outcome based on this correlation. Compared to previous studies, our study has several advantages. First, apart from the PPI network and the gene expression profiles, the DDI network and the somatic mutations within domains were integrated into our predictive model, which has improved the prediction performance to an accuracy of 88.3%, sensitivity of 87.2% and specificity of 88.9%. Second, our results compiled a list of cancer-associated gene signatures and domains, which provided testable hypotheses for further experimental investigation. Third, our approach is not specific to a specific cancer dataset and can thus be applied to different independent cancer data sets.

## Results

### Parameter tuning and validation on breast cancer data

**Figure 2:**
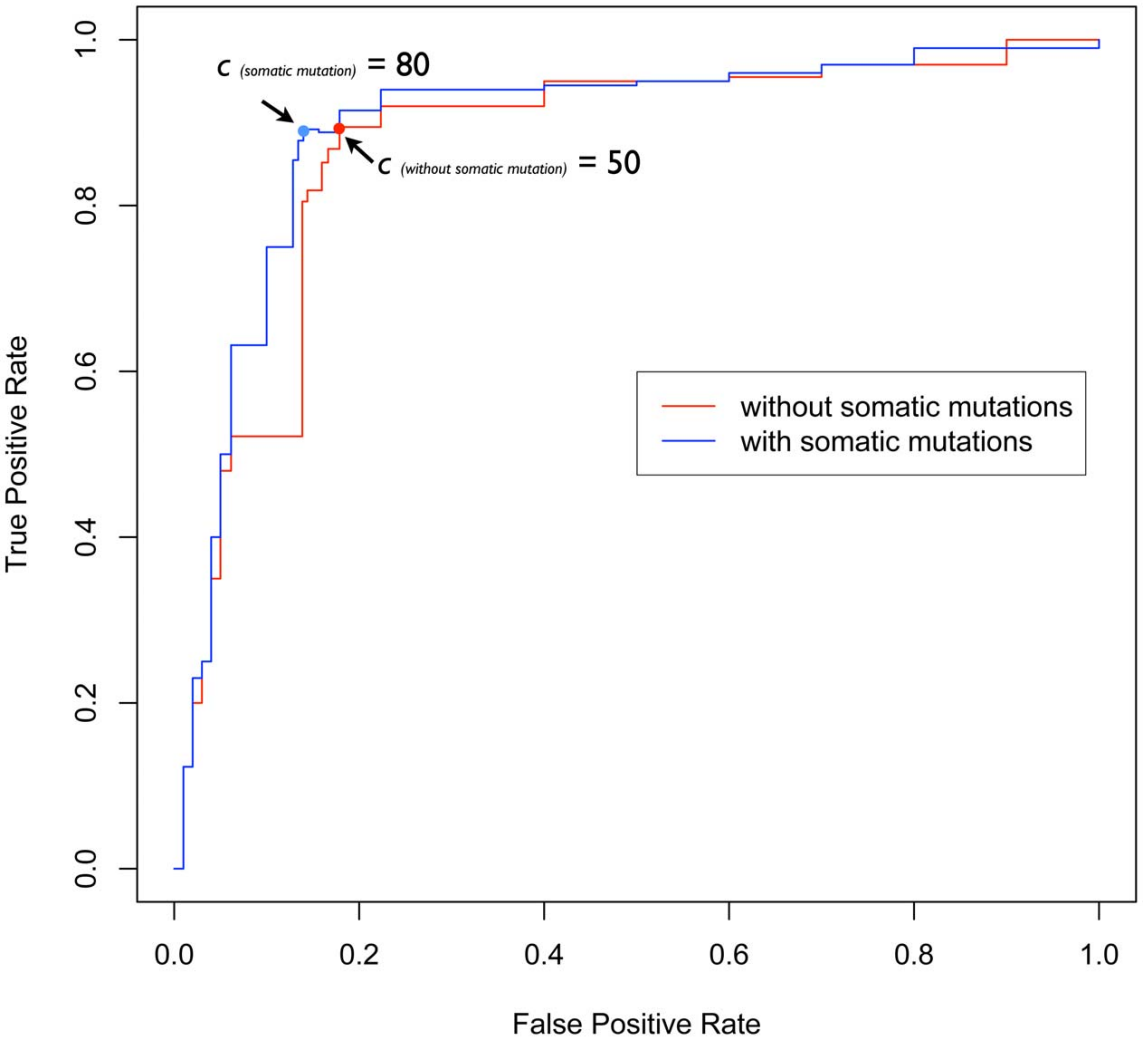
The performance of our approach using different thresholds of domain index scores (S_d_). Curve of receiver operating characteristics (ROC) plotted for different thresholds when our approach was tested against the breast cancer data set incorporating somatic mutation data and without incorporating somatic mutation data. The area under the curve (AUC) plotted for without somatic mutations and with somatic mutations is 0.854 and 0.892, respectively.

**Figure 3:**
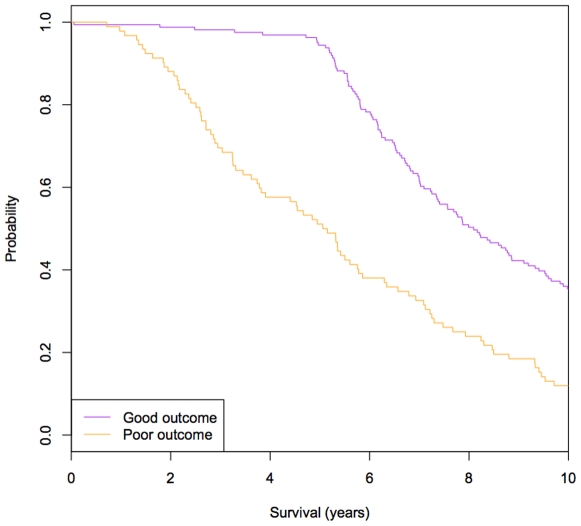
The ability of our approach to predict survival between two groups of breast cancer patients. The Kaplan-Meier survival plot for disease-free survival are shown for two group (“Good outcome” vs. “Poor outcome”) of breast cancer patients. The difference between two groups is statistically significant for 10-year survival at the P-value of 2.19×10^−8^ by the Mantel-Cox Log Rank test.

We tested whether our identified gene signatures are good indicators to differentiate a set of two groups of sporadic and non-familial breast cancer patients [25]. We defined patients who were disease free after extended follow-up as patients with ‘good outcome’ and those who died of disease as patients with ‘poor outcome’. The patient data was filtered to remove patients that were still alive with disease or dead from other reasons, as reported by Taylor [12]. The resultant dataset contained 179 patients with ‘good outcome’ and 74 patients with ‘poor outcome’. For each patient, a profile was computed based on the difference of the gene expression value between the gene signatures and their neighboring proteins. For the identification of gene signatures, we applied a scoring procedure to the protein domains present in each gene products based on the number of mutually exclusive DDIs they participated in (see methods). Using this approach we found that only one parameter needed to be tuned: the threshold (*c*) of domain index scores (*S_d_*). The threshold (*c*) was tuned by testing our approach on the breast cancer data set using different *S_d_* values (see methods). We then evaluated the performance of our approach by calculating three performance measurements: accuracy, sensitivity and specificity. In this study, accuracy = (TP+TN)/(TP+FP+TN+FN); sensitivity = TP/(TP+FN); specificity = TN/(TN+FP). A true positive is defined as the case that a “poor outcome” patient was successfully predicted as having the “poor outcome” and a true negative is defined as the case a “good outcome” patient was correctly predicted as having the “good outcome”. From the observation of the performance plot based on different *S_d_* (Figure 2), we concluded that our approach achieved the best performance with the accuracy of 85.8%, the sensitivity of 87.1% and the specificity of 82.6% when the threshold (*c*) of domain index scores (*S_d_*) were set as 50. We also found that with higher threshold (*c*), a smaller set of gene signatures were generated, and consequently lower the performance was. On the contrary, with lower threshold (*c*), the gene signature list contained higher noise and generated more false positives and negatives. Next, we did survival analysis to prove the ability to predict survival of our approach under this setting and observed the significantly different 10-year survival (Mantel-Cox Log Rank test, nominal P-value = 2.19×10^−8^) (Figure 3) between two groups of patients.

### The identified biomarkers might be involved in carcinogenesis

**Figure 4:**
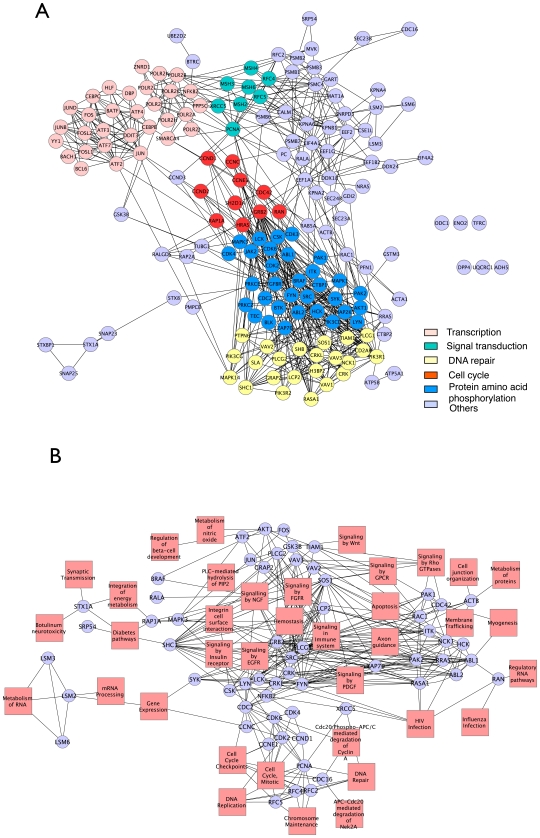
The biological functions of identified biomarkers. (A) The biological network of 171 gene signatures identified in the breast cancer data set using our approach. Each gene is labeled as different colors based on it biological function annotation derived from its gene ontology terms. (B) The pathway organization of identified gene signatures involved in 36 human biological pathways when they were mapped to the Reactome database [27].

A total of 171 gene signatures were identified in a breast cancer data set [25] using our approach at the threshold (*c*) of 50 as described in the above section. These gene signatures mainly are involved in 5 major cancer-related biological processes: transcription (P-value = 9.3×10^−10^), DNA repair (P-value = 3.8×10^−5^), signal transduction (P-value = 7.9×10^−13^), cell cycle (P-value = 1.1×10^−9^) and protein phosphorylation (P-value = 2.9×10^−26^) if we performed GO Term enrichment analysis using FuncAssociate [26] (Figure 4A). The complete list of over-represented GO terms associated with identified gene signatures is in the supplementary materials (Table S1). In addition, 36 human biological pathways can be derived when we mapped the gene signatures to the Reactome database that contains manually curated human biological pathways [27] (P-value<0.001) (Figure 4B). For instance, the well-known oncogenic transcription factors such as FOS, JUN and NFκB were identified as gene signatures by this study. We also identified some DNA repair genes including XRCC5, MSH, PCNA and others as gene signatures. These genes were demonstrated to cause cancer because mutations in those genes disable the ability of DNA repairing, which subsequently leads to the accumulation of mutations [28], [29], [30]. Genes involved in signal transduction, an important type of pathways in cancer development, such as MARK14, VAV1 and PIK3R1 were also identified as gene signatures in this study. Besides, a group of cyclin-dependent kinases (CDK2, CDK3, CDK4, CDK6) that control cell proliferation [31] and genes (SRC, ABL1) related to protein phosphorylation [32] were also identified by our approach. In summary, there were 38% (65 out of 171) of the identified gene signatures found to be the genes associated with cancers in Online Mendelian Inheritance in Man (OMIM; http://www.ncbi.nlm.nih.gov/omim/). This percentage is significantly greater than what could be found purely by chance (Adjusted P-value<10^−12^, by Fisher's Exact Test), indicating the capability of our approach to identify disease genes. Interestingly, only 15% (26 out of 171) of the identified gene signatures were known cancer susceptibility genes compared to a list of 410 genes downloaded from The Cancer Gene Census (http://www.sanger.ac.uk/genetics/CGP/Census/), whose mutations had been causally implicated in cancer, but the small overlap is still statistically significant at P-value of 7.7×10^−6^ by Wilcoxon Test. This result was consistent with those of the previous studies, which yielded 21% and 16%, respectively [12], [14]. In order to examine the importance that the cancer susceptibility genes contribute to cancer prognosis, we employed these 410 known cancer susceptibility genes as signature genes to predict breast cancer outcomes, we observed a relatively low accuracy of 72.6%, sensitivity of 72.9% and specificity of 71.4% if tested on the same breast cancer set (Figure S1). Taken together, the low percentage of known cancer susceptibility genes present in our gene signature list suggests that the mutations in not only these genes, but also other genes, might collectively affect the process of tumor-aggressiveness and response to therapy in various ways by disrupting the modularity of the PPI network. Among other genes in our gene signature list but not in the list of known cancer susceptibility genes, 32% (46 out of 145) of genes can be mapped to the human biological pathways in which known cancer susceptibility genes anticipate in the Reactome database (P-value = 2.1×10^−8^ by Z-test). Therefore, we speculated that the other genes could be the downstream effectors of the cancer susceptibility genes and the changes in their expression value could reflect the disruption of the PPI network caused by the mutations in the cancer susceptibility genes. In order to investigate what types of domains tend to exist in ‘singlish-interface’ proteins and disrupt protein interactions, we calculated the number of involved domain-domain interactions of each domain in ‘singlish-interface’ proteins against the whole genome and compared it to that expected by chance (P<0.01, Z-test) (see Figure 1). We identified a list of 29 over-represented domains within 171 gene signatures (Table 1). Interestingly, 93% (27 out of 29) of the domains were annotated as cell signaling domains such as SH2, Pkinase and Ras according to the SMART database [33] indicating that these domains were likely to play a critical role in carcinogenesis through disrupting the protein interactions within signaling pathways. For example, the SH2 domain of the oncoprotein Src interacts with 86 domains within 57 proteins. It has been demonstrated that SH2 domain regulates intracellular signalling cascades by interacting with high affinity to phosphotyrosine-containing target peptides [34], [35] and is related to cancer cell migration and proliferation [36]. Another example is that the Pkinase domain contains the catalytic function of protein kinases that are essential in the process of phosphorylation [37], [38]. Many diseases including cancer are caused by dysfunction of phosphorylation [39].

**Table 1:** A list of over-represented domains within gene signatures.

Domain	Name	DDIs	P-value
PF00017	SH2	86	1.63E-24
PF00018	SH3_1	70	1.39E-23
PF00069	Pkinase	49	1.41E-23
PF00071	Ras	45	2.13E-23
PF00170	bZIP_1	42	2.49E-23
PF07716	bZIP_2	34	2.97E-23
PF00130	C1_1	23	5.56E-23
PF00271	Helicase_C	23	6.34E-23
PF00270	DEAD	23	7.18E-23
PF00169	PH	22	7.21E-23
PF00096	zf-C2H2	22	9.03E-23
PF05739	SNARE	21	9.94E-23
PF00023	Ank	20	9.98E-23
PF01833	TIG	20	1.44E-22
PF00433	Pkinase_C	19	2.17E-22
PF00004	AAA	18	9.83E-22
PF01423	LSM	17	1.04E-21
PF00786	PBD	16	3.90E-21
PF00134	Cyclin_N	14	4.60E-17
PF00022	Actin	14	9.84E-17
PF00804	Syntaxin	13	1.80E-16
PF00595	PDZ	12	2.96E-15
PF00125	Histone	12	3.37E-14
PF00617	RasGEF	11	9.15E-12
PF00618	RasGEF_N	11	5.99E-11
PF05192	MutS_III	11	6.15E-10
PF00621	RhoGEF	10	6.40E-10
PF00515	TPR_1	10	6.73E-06
PF02984	Cyclin_C	10	7.19E-06

The first two columns are Pfam domain ID and name. The third column is the number of involved domain-domain interactions of each domain within gene signatures against the whole genome and then compared it to that expected by chance using Z-test (P-value in the fourth column).

### Knowing which somatic mutations are present increases the accuracy of our approach

It is widely accepted that genetic changes such as somatic mutations are implicated in cancer development [40]. Also, some somatic mutations reveal the role of functional domains in hereditary disorders and complex diseases [41]. For example, tumors highly sensitive to epidermal growth factor receptor (EGFR) tyrosine kinase inhibitors often contain dominant mutations in exons that encode a portion of the tyrosine kinase (TK) domain of EGFR [42]. To investigate the possibility that somatic mutations within domains represent another type of important signal to differentiate two classes of patients, we incorporated the somatic mutation data compiled from the COSMIC database to our scoring model (see methods) by searching for the genes having mutually exclusive domains that harbor somatic mutations. We hypothesized that these mutations could disrupt DDIs and PPIs and consequently change the modularity of the human protein interaction network. By employing the modified domain index function that incorporates the somatic mutation data, we tuned again the threshold *(c)* using different *S_d_* values. At the threshold of S_d_ = 80, our approach identified 126 gene signatures and achieved the accuracy of 88.3%, the sensitivity of 87.2% and the specificity of 88.9% when tested on the breast cancer outcome data (Figure 2). All of 126 gene signatures belong to a list of 171 gene signatures identified by the CAERUS approach without integrating the somatic mutation data, which indicates that 45 gene signatures failed to pass a preset threshold after the somatic mutation data were used. To test weather the slight improvement on predictive performance (0.038 difference in the area under the ROC curve) is statistically significant, we tested CAERUS on randomized 126 genes from the list of 171 gene signatures and repeated this procedure 100 times (Figure S2). We found that this improvement is indeed statistically significant at the P-value of 2.8×10^−5^ by Wilcoxon Test. Compared to the performance of CAERUS' that does not incorporate the somatic mutation data, the improvement on CAERUS' performance by integrating the somatic mutation data suggests that the somatic mutation data can be used to supplement our accuracy to predict cancer survival outcome. However, the capability of using the mutation data appears limited due to the fact that not all mutations are driving the development of the cancer, the so-called “driver mutations” [43]. Minor performance improvement could be explained by the incompleteness of currently available somatic mutation data or the bias introduced by “passenger mutations”. With the help of the numerous Cancer Genome Projects [44], [45], the size of the somatic mutations data in human will grow in the near-future possibly providing us with even better indications from mutation data.

### Using gene expression, and the comparison with other approaches

**Figure 5:**
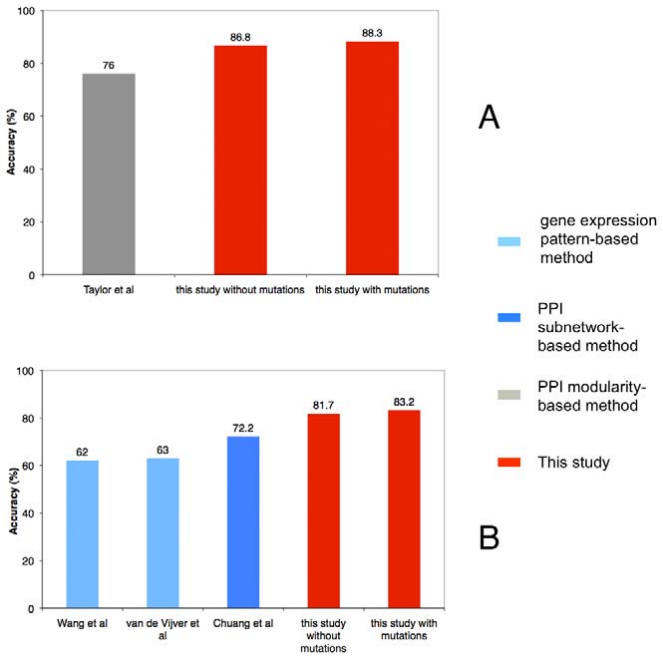
Predictive performance comparison between different approaches. (A) Our approach was applied on the same data set as Taylor et al [12]. Compared to the predictive performance of Taylor et al, our approach achieved better accuracy of 83.2% (with somatic mutation data) and 81.7% (without somatic mutation data). (B) Our approach was applied on the same data set as Chuang et al [14], Wang et al [10] and van de Vijver et al [25] and achieved better accuracy of 83.2% (with somatic mutation data) and 81.7% (without somatic mutation data) compared to other three approaches (Chuang et al with accuracy of 72.2%, Wang et al with accuracy of 62% and van de Vijver et al with accuracy of 63%).

Identifying novel prognostic markers to classify different cancer outcomes has been widely studied with the increasingly available gene expression profiles. The approaches described in previous publications can be categorized into three classes: 1) gene expression pattern-based method, in which markers are selected based on whether their expression profiles can differentiate different groups of patients [9], [10]; 2) PPI subnetwork-based method, in which each marker represented as a subnetwork in the PPI network was identified by maximizing the mutual information measuring the association between the expression value of each gene in the subnetwork and the types of patients [14]; 3) PPI modularity-based method in which each gene signature was identified by comparing the difference of the gene expression value between a hub gene and their interacting partners in the PPI network [12]. In this study, we employed a novel approach based on finding genes in the PPI network with mutually exclusive domains and somatic mutations located in these domains as the markers. Wang et al [10] and van de Vijver et al [25] reported 63% and 62% accuracy, respectively, for the prediction of metastasis using gene expression pattern-based methods. Using the PPI subnetwork-based method, Chuang et al [14] yielded the accuracy of 72.2% and 70.1% using the same data set as Wang et al and van de Vijver et al did. Using the PPI modularity-based method, Taylor et al [12] reported the accuracy of 76% tested on the breast cancer patient data set. We first applied our approach on the same data set as Chuang et al [14] used and adopted the identical training and testing strategy (five-fold cross-validation) and observed that our approach achieved the accuracy of 83.2%, the sensitivity of 84.6% and the specificity of 82.5%. Next, we applied our approach on the same data set as Taylor et al [12] used and adopted the identical training and testing strategy (five-fold cross-validation) and observed that our approach achieved the accuracy of 87.3%, the sensitivity of 87.2% and the specificity of 88%, which indicates that our method outperforms other approaches and provides a promising solution to predict cancer outcome (Figure 5).

### The robustness of our approach

**Figure 6:**
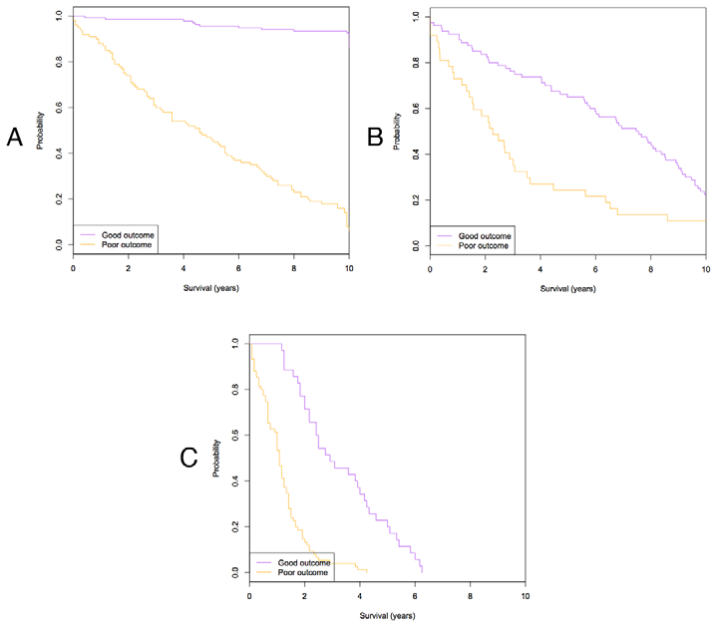
The ability of our approach to predict survival between two groups of breast cancer patients using different independent data sets. (A) The Kaplan-Meier survival plot for disease-free survival is shown for good or poor prognostic groups derived from an independent breast cancer date set from Miller et al. [46]. The difference between two groups is statistically significant for 10-year survival at the P-value of 1.8×10^−25^ by the Mantel-Cox Log Rank test. (B) The Kaplan-Meier survival plot for disease-free survival is shown for good or poor prognostic groups derived from another breast cancer independent date set from Chin et al. [47]. The difference between two groups is statistically significant for 10-year survival at the P-value of 7×10^−4^ by the Mantel-Cox Log Rank test. (C) The Kaplan-Meier survival plot for disease-free survival is shown for good or poor prognostic groups derived from an ovarian cancer data set from Yoshihara et al. [48]. The difference between two groups is statistically significant for 10-year survival at the P-value of 3.12×10^−12^ by the Mantel-Cox Log Rank test.

To test the robustness of our approach on different independent data sets or different types of cancer, we first applied our approach to a data set that included 236 primary invasive breast tumors [46]. Using five-fold cross-validation, our approach achieved the accuracy of 92.4%, the sensitivity of 94% and the specificity of 90.2%. Our approach also revealed significantly different 10-year survival (Mantel-Cox Log Rank test, nominal P-value = 1.8×10^−25^) (Figure 6A). Another independent data set that includes 117 primary breast tumors was utilized to evaluate the performance of our approach [47]. Using the leave-one-out cross-validation (LOOCV) strategy due to insufficient sample size, our approach achieved the accuracy of 89.8%, the sensitivity of 85.7% and the specificity of 91.6% with the significantly different 10-year survival (Mantel-Cox Log Rank test, nominal P-value = 7×10^−4^) (Figure 6B). These results indicate that our predictive approach has good performance in predicting breast cancer outcome when tested on different independent data sets. Next, we compiled a set of 110 patients with advanced-stage ovarian cancer that contains the gene expression profiles of 34 patients without disease recurrence and 76 patients with disease recurrence [48]. We applied our approach to this data set using the five-fold cross-validation strategy. We observed that our approach achieved the accuracy of 90.1%, the sensitivity of 90.4% and the specificity of 88.6%, further validating the robustness of our predictive approach when tested on different types of cancer data sets. The good predictive performance is also demonstrated by the 10-year survival curve (Mantel-Cox Log Rank test, nominal P-value = 3.12×10^−12^) (Figure 6C).

## Discussion

Biological network information has been proven to be a useful feature to improve prognosis performance [12], [14]. In this context, our study constitutes the first predictive method to classify cancer outcomes based on the information of protein interaction interfaces in the protein interaction networks. Compared to previous predictive approaches, the most outstanding feature of CAERUS is that we investigated biological network disruptions linked to cancer outcomes at the protein domain level. The favorable predictive performance of our approach suggests that association exists between cancer outcome and the alteration in the protein interaction network, and more importantly, that the alteration is probably caused by the genetic variations within interacting domains. These genetic variations are capable of interrupting the physical interactions between proteins and thus causing abnormal biological functions associated with cancer progression. In this study, we applied CAERUS primarily on breast cancer data sets and achieved favorable predictive performance. However, the strength of CAERUS is not restricted to a certain type of cancer; other types of cancer such as ovarian cancer can be analyzed in a similar manner. It is worth noting that the potential of the approach described in this study is restrained by the limitations of currently available data sources, as these data sources, such as the protein interaction data, the domain interaction data, the gene expression data are incomplete and also contain biases. The currently available somatic mutation data is also limited and not individual-based. With the growth in the size and better quality of these data sets, our study would lead to a more reliable and robust prognosis tool to access cancer outcome. Furthermore, this study could be optimized with the integration of additional types of data. For instance, we could achieve better predictive performance by integrating the patients' transcriptome data obtained via the RNA-seq technology which measures gene expression levels more accurately compared to the microarray approach [49]. With patient-specific somatic data, it will become possible to fine-tune the CAERUS approach and we would be able to achieve better performance. In addition, the effects of protein post-translation modifications such as phosphorylation, methylation and acetylation could also be potentially integrated into our model to reflect the influence of these types of modifications on the organization of the protein-protein interaction network during cancer development. In conclusion, we presented a novel and integrated approach to predict different cancer outcomes, which could be of significant clinical application.

## Materials and Methods

### Data set collection

We downloaded 108,307 unique PPIs in human from the iRefIndex database (ftp://ftp.no.embnet.org/irefindex/data) version of June 4, 2009. The iRefIndex database [50] provides a non-redundant list of protein interactions derived from several major protein interaction databases including BIND, BioGRID, CORUM, DIP, HPRD, IntAct, MINT, MPact, MPPI and OPHID. We also used a set of DDIs downloaded from the iPfam database [51], a DDI database based on RCSB Protein Data Back (PDB) crystal structures (http://www.pdb.org), which consists of 3,020 DDIs and 914 domains. For somatic mutations involved in cancer, a list of 88,641 somatic mutations was retrieved from the COSMIC database (version 43) that contains the mutation data and associated information extracted from the primary literature [52].

A set of gene expression profiles of 295 breast cancer patients and clinical results was collected from the work of van de Vijver and colleagues [25]. This data set was applied to test the performance of CAERUS. We defined patients who were disease free after extended follow-up as patients with ‘good outcome’ and those who died of disease as patients with ‘poor outcome’. The data was filtered to remove patients that were still alive with disease or dead from other reasons, as reported by Taylor [12]. The resultant dataset contained 179 patients with ‘good outcome’ and 74 patients with ‘poor outcome’. The mean duration of follow-up was 7.5 years for ‘good outcome’ patients and 2.8 years for ‘poor outcome’ patients. Two independent breast cancer data sets were employed for the validation purpose. The first data set consists of gene expression profiles of 236 patients with primary invasive breast tumors that derived from oligonucleotide arrays and the corresponding survival data of these patients were collected based on the patient records accompanying with the paper [46]. In this data set, 134 patients were classified as ‘good outcomes’ and 102 patients with ‘bad outcomes’ using the same abovementioned criteria. The mean duration of follow-up was 10.9 years for ‘good outcome’ patients and 4.9 years for ‘poor outcome’ patients. The second data set was obtained from the gene expression profiles of a cohort of 117 patients with breast tumors, of which 83 patients had ‘good outcomes’ and 34 patients had ‘bad outcomes’ derived from each patient’s survival duration and disease recurrence information included in the paper [47]. The mean duration of follow-up was 7.2 years for ‘good outcome’ patients and 2.1 years for ‘poor outcome’ patients. In addition, we compiled the data from a set of 110 Japanese patients who were diagnosed with advanced-stage serous ovarian cancers [48]. The gene expression profiles and the clinical characteristics of each patient were extracted from the supporting materials of the paper, in which 34 patients were labeled as ‘good outcomes’ and 76 patients as ‘bad outcomes’ using the same criteria described in previous data sets. The mean duration of follow-up was 3.3 years for ‘good outcome’ patients and 1.2 years for ‘poor outcome’ patients.

### Gene signature finding algorithm

#### Step A

We have a query network X comprised of proteins {x_1_, …, x_n_} and known PPIs between x_i_ and x_j_ from the iRefIndex database. For each protein *x_i_* in the query PPI network, we have a mapping function D(x_i_) = {d_1_, …, d_n_} that returns the set of annotated domains of this protein according to the Pfam database. Here, d_i_ are the individual domains.

#### Step B

For each domain *d_i_* in the domain set D(x_i_), we counted the number of domain pairs on aggregate between *d_i_* and a set of domains of neighboring/interacting proteins *neighbor[x_i_]* represented in the interacting domain-domain pairs previously established in the iPfam database.

#### Step C

A domain index score was assigned to each protein in the query PPI network by the following equation:
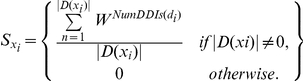
where *NumDDIs(d_i_)* is the number of DDIs of between *d_i_* and a set of domains of neighboring/interacting proteins as calculated by the Step B. Here, W is an exponential function at the base of 2, which meant that we add weights exponentially to a domain if it has multiple DDIs. In order to take it into account that somatic mutations occur within domains, we used to a modified domain index function to calculate scores to each protein:
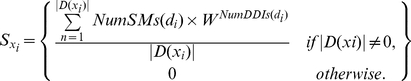
where *NumSMs(d_i_)* is the number of somatic mutations of *d_i_*.

#### Step D

For each protein x_i_, if the domain index score was over the preset threshold *c*, this protein was regarded as a gene signature and was utilized for the neighboring gene expression analysis. The threshold *c* was tuned by performing a modified five-fold cross-validation strategy in which we firstly adopted the leave-one-out cross-validation (LOOCV) strategy for different 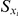 using 80% of the original data set (expression profiles), and then used the discovered value *c* to validate against the remaining data set (20%). This procedure was repeated 5 times in a manner that each data point (a gene expression profile) in the dataset was used once as the validation data.

### Calculation of neighboring gene expression profiling score

Given a gene expression data set and a gene signature *x*, we computed a score to measure the difference in co-expression of the gene signature and its neighboring proteins P = {p_1_, …., p_n_) in the PPI network between two types of cancer outcomes (“good/disease-free” vs. “poor/recurrent disease”) using the following equation:
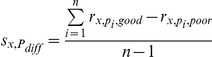
where *n* is the number of interactors of the gene signature *x*; *r_x,pi,good_* and *r_x,pi,poor_* is the Pearson correlation coefficient of expression values of protein x and its interactors P = {p_1_, …., p_n_} in different groups of patients (good or poor). The Pearson correlation coefficient of expression values of protein *x* and its interactors in the different groups is calculated by the following equation:
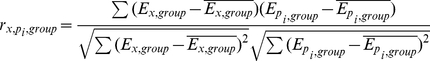



### Construction of the naïve Bayes classifier

As a probabilistic model based on the Bayes' theorem, the naïve Bayes classifier has been widely applied to the classification problem in different fields of the biological sciences such as inferring cellular networks [53], modeling protein signaling pathways [54] and the prediction of protein-protein interaction interfaces [55]. Given the training dataset and testing dataset in which each data sample is represented as an n-dimensional vector (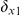, 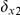 …, 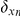), 2 classes (C_good_, C_poor_). Here, n is the number of gene signatures; 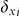 is the difference in co-expression of the gene signature *i* and its neighboring proteins in the PPI network in patient *x*. The prediction procedure follows as:

According to the Bayes theorem, we can get the highest posterior probability of each cancer patient sample x based on the following equation:
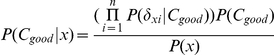
where the class prior probabilities P(C_good_) is calculated by X_good_/X, the value of the number of training samples of class C_good_ divided by the total number of training sample. P(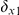
|C_good_), P(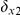
|C_good_), …, P(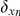
|C_good_) can be easily calculated by 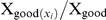, where 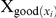 is the number of training samples of class C_good_ having the gene expression difference score 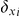 falling into one certain bin/category, and X_good_ the number of training samples belonging to C_good_. In this study, we divided the gene expression difference score into 20 bins as it ranges from 0 to 1.

In order to classify cancer patient samples in the testing dataset, we calculated the P(x|C_i_)P(C_i_) for each class C_i_. Sample/patient x was then predicted as belonging to class C_good_ if and only if
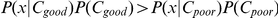
In other words, it is assigned to the class C_good_ for which P(x|C_good_)P(C_good_) is the maximum.

### Availability

The method has been implemented in Perl and is available for downloading from http://www.oicr.on.ca/research/ouellette/caerus. It is distributed under the terms of GPL (http://opensource.org/licenses/gpl-2.0.php)

## Supporting Information

Figure S1 The performance of our approach using 410 known cancer susceptibility genes as gene signatures. Curve of receiver operating characteristic (ROC) plotted for different thresholds when our approach was tested against the breast cancer data set incorporating somatic mutation. The area under the curve (AUC) is 0.726.
(0.44 MB TIF)

Figure S2 The distribution of the predictive performance of our approach using different random gene signature sets. CAERUS was tested on randomized 126 genes from the list of 171 gene signatures and this procedure was repeated 100 times. Histogram of the area under the curve (AUC) values was plotted for 100 runs. Red vertical bar represents the AUC value of using 126 gene signatures identified by incorporating the somatic mutation data set.
(0.65 MB TIF)

Table S1 A list of 222 over-represented GO terms associated with identified gene signatures.
(0.09 MB XLS)
