# Supplementary figures and images for: CAERUS: Predicting CAncER oUtcomeS Using Relationship between Protein Structural Information, Protein Networks, Gene Expression Data, and Mutation Data
Source: PLoS Comput Biol. 2011 Mar 31;7(3):e1001114. doi: 10.1371/journal.pcbi.1001114 (PMC3068924; doi:10.1371/journal.pcbi.1001114)

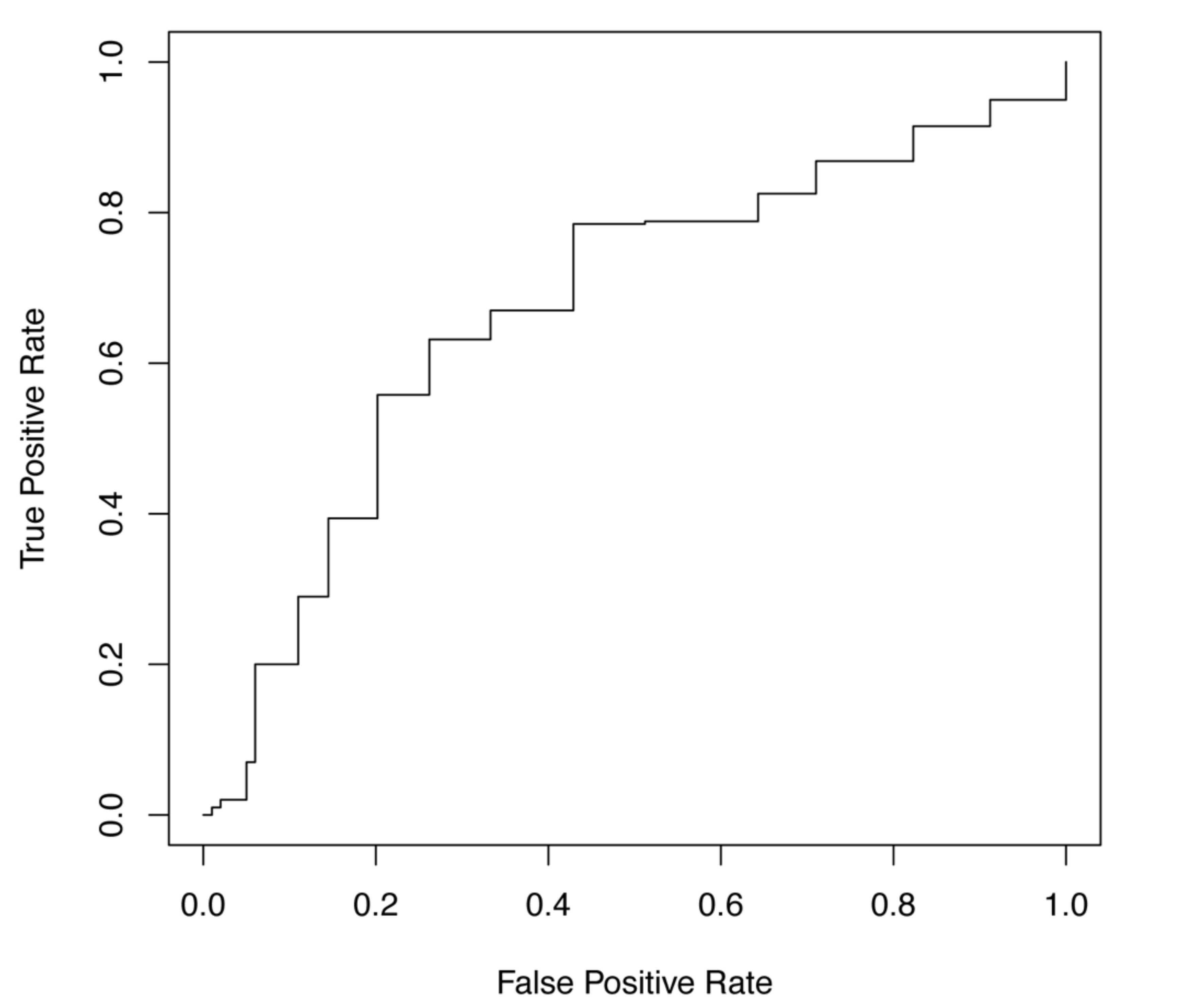

Supplement: Figure S1 — The performance of our approach using 410 known cancer susceptibility genes as gene signatures. Curve of receiver operating characteristic (ROC) plotted for different thresholds when our approach was tested against the breast cancer data set incorporating somatic mutation. The area under the curve (AUC) is 0.726. (0.44 MB TIF) [file pcbi.1001114.s001.tif]

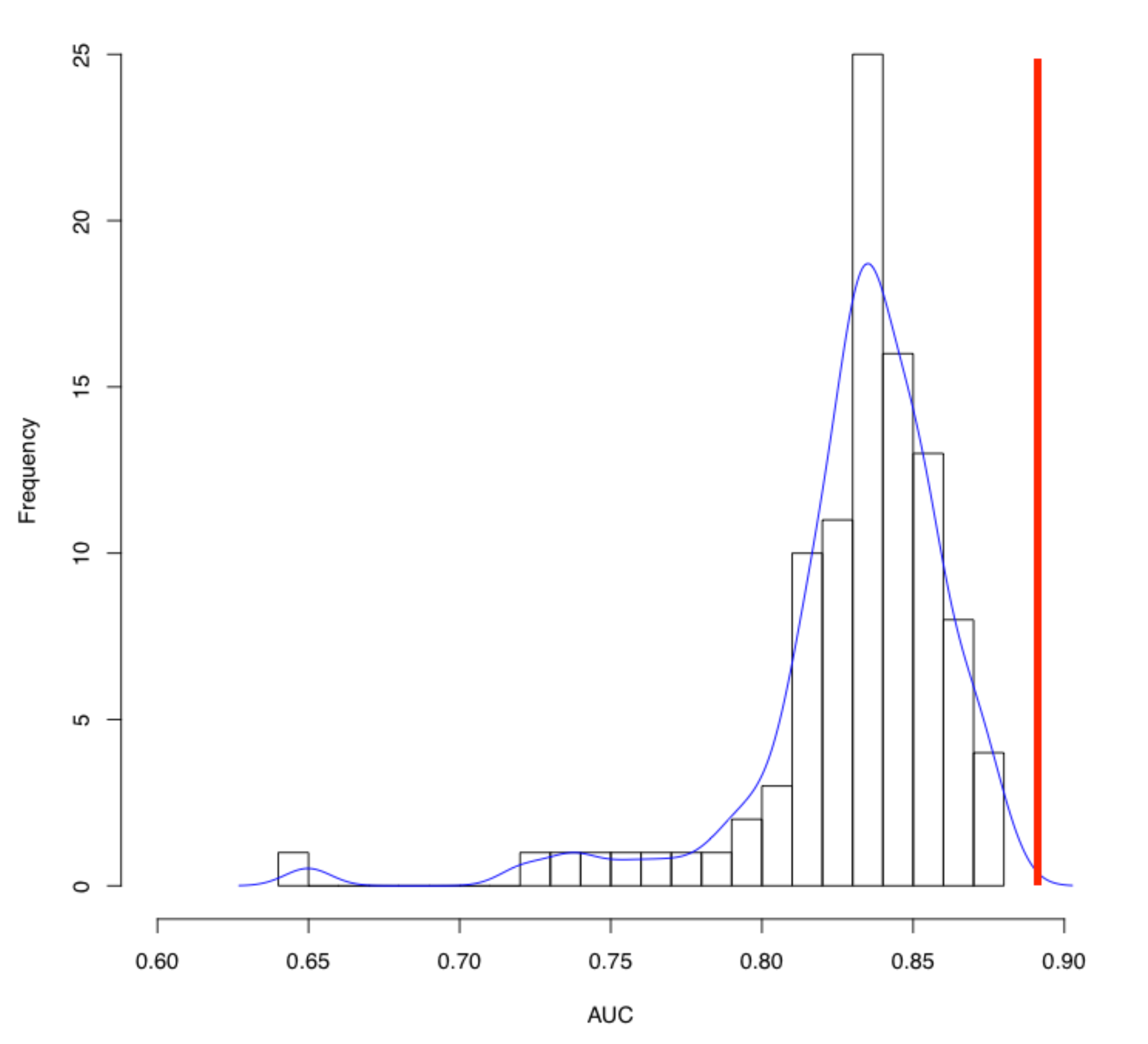

Supplement: Figure S2 — The distribution of the predictive performance of our approach using different random gene signature sets. CAERUS was tested on randomized 126 genes from the list of 171 gene signatures and this procedure was repeated 100 times. Histogram of the area under the curve (AUC) values was plotted for 100 runs. Red vertical bar represents the AUC value of using 126 gene signatures identified by incorporating the somatic mutation data set. (0.65 MB TIF) [file pcbi.1001114.s002.tif]
